# M-CSF and GM-CSF Regulation of STAT5 Activation and DNA Binding in Myeloid Cell Differentiation is Disrupted in Nonobese Diabetic Mice

**DOI:** 10.1155/2008/769795

**Published:** 2009-01-20

**Authors:** B. Rumore-Maton, J. Elf, N. Belkin, B. Stutevoss, F. Seydel, E. Garrigan, S. A. Litherland

**Affiliations:** ^1^College of Veterinary Medicine, University of Florida, 2015 SW 16th Avenue, Gainesville, FL 32610, USA; ^2^Department of Pathology, Immunology, and Laboratory Medicine, College of Medicine, University of Florida, P. O. BOX 100275, 1600 SW Archer Road, Gainesville, FL 32610, USA; ^3^Burnham Institute for Medical Research-Lake Nona, SLSL Buliding M6-1025, Rm 102-9, Kennedy Space Center, Lake Nona, FL 32899, USA

## Abstract

Defects in macrophage colony-stimulating factor (M-CSF) signaling disrupt myeloid cell differentiation in nonobese diabetic (NOD) mice, blocking myeloid maturation into tolerogenic antigen-presenting cells (APCs). In the absence of M-CSF signaling, NOD myeloid cells have abnormally high granulocyte macrophage colony-stimulating factor (GM-CSF) expression, and as a result, persistent activation of signal transducer/activator of transcription 5 (STAT5). Persistent STAT5 phosphorylation found in NOD macrophages is not affected by inhibiting GM-CSF. However, STAT5 phosphorylation in NOD bone marrow cells is diminished if GM-CSF signaling is blocked. Moreover, if M-CSF signaling is inhibited, GM-CSF stimulation *in vitro* can promote STAT5 phosphorylation in nonautoimmune C57BL/6 mouse bone marrow cultures to levels seen in the NOD. These findings suggest that excessive GM-CSF production in the NOD bone marrow may interfere with the temporal sequence of GM-CSF and M-CSF signaling needed to mediate normal STAT5 function in myeloid cell differentiation gene regulation.

## 1. INTRODUCTION

Myeloid cell differentiation gives rise to 3 populations of professional antigen-presenting cells (APC) in the immune system: monocytes, macrophages, and dendritic cells (DC). Sequential signaling from interleukin-3 (IL-3), granulocyte-macrophage-, macrophage-, and granulocyte-colony stimulating factors (GM-, M-, and G-CSF, respectively) sets the microenvironment necessary for bone marrow myeloid precursor cells to differentiate and function [1]. The timing and level of each cytokine in myeloid cell microenvironmentare tightly controlled in order for myeloid cells differentiate to functional APC, and to prevent the premature activation of genes involved in macrophage activation, such as cyclooxygenase/prostaglandin synthase 2 (COX 2/PGS2), which could promote deleterious inflammation.

The cytokine GM-CSF has a unique dual influence in myeloid cells, acting first as a differentiation factor in myeloid hematopoiesis, and then later as an activation stimulus in mature monocytes and macrophages. Both overexpression and knockout deletion of GM-CSF in mice can lead to dysregulation of myeloid differentiation and autoimmune disease [2], suggesting that GM-CSF influence exerts discrete temporal and quantitative regulatory effects in myeloid cell differentiation and mature cell activation. This switch in GM-CSF function relies heavily on a change in responsiveness of cells to GM-CSF before and after M-CSF stimulation [3], and on the ability of GM-CSF to activate STAT5 phosphorylation at different stages of myeloid cell maturation [4, 5].

Work by Piazza et al. [4] suggests that STAT5 proteins can act as key cytokine and growth factor-inducible regulatory “switches” for gene expression during myeloid differentiation and activation. In early myeloid differentiation stages, IL-3 and GM-CSF can induce signaling through both STAT5A (94-96kD) and B (94-92kD) isoforms which act as adaptor molecules for histone acetylases and deacetylases [4–7]. Subsequent signaling through M-CSF and G-CSF signals in matured and activated cells also activates STAT5 [4, 8, 9]. Thus, during cytokine-induced differentiation, STAT5 can act to promote both gene transcriptional regulation through epigenetic chromatin dynamics [4, 6, 7].

 Incomplete myeloid differentiation is emerging as a common component of multiple autoimmune pathologies, both in mice and man. Serreze et al. (1993) [10] found that myeloid antigen presenting cell (APC) differentiation is impaired in NOD mouse bone marrow due to a lack of responsiveness to M-CSF. This nonresponsiveness was not linked to any defect in M-CSF expression or defect in its receptor, but to unknown problems with M-CSF intracellular signaling. Morin et al. (2003) [11] also noted that high GM-CSF can skew NOD myeloid differentiation away from macrophage and DC development, and toward an excess of granulocyte production. Several laboratories have reported disproportion of immature DC compared to mature DC in the diabetic NOD, a process dependent on regulation of GM-CSF signaling [10–13]. These dysfunctional APC phenotypes suggest that NOD myeloid cell differentiation and activation are defective at a point or points, where the determination of specific cell lineage decisions is made based on the cytokine microenvironment [1, 4]. Similar defective myeloid cell phenotypes have been seen in human autoimmune diseases such as T1D, SLE, and autoimmune thyroid diseases (ATD) [10–15]. The result of these defective myeloid cell properties is a loss of professional APC that can efficiently promote tolerance and regulate appropriate macrophage inflammatory responses [12, 16, 17]. Loss of these functions sets up an immune microenvironment conducive to immunopathogenesis.

In our studies of APC dysfunction in autoimmunity, we have found that both NOD and autoimmune human myeloid cells express abnormally high levels of GM-CSF and have prolonged STAT5 activation after even brief exposure to GM-CSF [18–20]. In these studies, we test the potential connection of GM-CSF signaling and its activation of STAT5 isoforms with the defect in M-CSF signaling in NOD myeloid differentiation *in vitro*. We find that both GM-CSF overproduction and persistent STAT5 phosphorylation phenotypes are amplified in autoimmune NOD bone marrow cells, but can be overridden by *in vitro* inhibition of endogenous GM-CSF signaling. Furthermore, the persistent STAT5 phosphorylation phenotype seen in NOD myeloid cells can be recreated in non-autoimmune C57BL/6 bone marrow cultures by blocking M-CSF while stimulating with NOD level GM-CSF. These findings *in vitro* suggest that the defect in NOD M-CSF signaling *in vivo may* occur during the switch of influence between GM-CSF to M-CSF during myeloid differentiation, and indicate that prolonged expression of GM-CSF promotes the dysregulation of STAT5, which may interfere with M-CSF signaling during NOD myeloid cell differentiation.

## 2. METHODS AND MATERIALS

### 2.1. Mouse strains used

Eight to twelve-week old NOD and C57BL/6 female mice (The Jackson Laboratory, Bar Harbor, Mich, USA) were used for all studies. These strains were maintained as breeding stock in our Pathology Department SPF colony, in microisolator cages with food and water ad libium. All procedures were conduced to maintain humane treatment of all animals according to IACUC approved protocols B083 and D754, and humanely euthanized by cervical dislocation while over-anesthetized. All tissue collections were done postmortem. A minimum of 3 animals per strain was used per treatment for each run of each experiment.

### 2.2. Bone marrow differentiation culture and sample preparation

Long bones from the legs were collected postmortem into cold RPMI + 10%FCS medium (Cellgro, MediaTech, Manassas, Va, USA) and the marrow flushed into a sterile tube by cold medium injected into the bone through a 30-gauge needle. The marrow samples were washed with cold media by centrifugation and red blood cells in samples lysed by incubation in nonisotonic 0.84% ammonium chloride buffer (Fisher Scientific, Pittsburgh, Pa, USA). The remaining bone marrow cells are then plated at 5–10 million cells/well on tissue culture dishes and fed with fresh sterile medium alone or with medium containing 1000 U/mL of GM-CSF (In vitrogen-Biosource, Carlsbad, Calif, USA) + 2 *μ*g/mL anti-M-CSF blocking antibodies (Pierce-Endogen, Rockford, Ill, USA), or medium containing 500 U/mL M-CSF (In vitrogen) + anti-GM-CSF blocking antibodies (Pierce-Endogen). Cultures were maintained for 24 hours at 37°C/5%CO_2_, then washed, and resupplemented for an additional 24 hours in culture at 37°C/5%CO_2_. Cells were then analyzed for myeloid phenotypic identification and STAT5 phosphorylation by flow cytometry and deconvolution microscopy.

### 2.3. Flow cytometric and deconvolution microscopic image analysis

Flow cytometric analysis of fluorescently conjugated antibody surface and intracellular binding was used to phenotypically identify myeloid cells and quantify the level of STAT5 tyrosine phosphorylation (STAT5Ptyr) in cells *ex vivo* and after *in vitro* stimulation. Cells were first labeled with anti-CD11b antibody FITC or PE conjugates (BD Biosciences, Calif, USA) to identify monocytes and macrophages in the sample, then fixed with 2% (v/v C_f_) formaldehyde (Sigma-Aldrich, St. Louis, Mo, USA) and permeabilized using 0.5% saponin (Sigma-Aldrich) in a high protein, isotonic FACS buffer [14]. Intracellular staining of STAT5Ptyr was done using FITC or APC-conjugated anti-STAT5Ptyr-specific monoclonal antibodies (Millipore-Upstate monoclonal antibody conjugated FITC (Millipore-Upstate) or with APC using labeling kit, Prozyme, San Leandro, Calif, USA). Data were analyzed as the percentage of positive myeloid cells (%STATPtyr+/CD11b+) to assess the number of cells in the myeloid population with activated STAT5. After flow analysis, the labeled cells are centrifuged onto slides and counterstained for chromatin (DAPI, Invitrogen-Molecular Probes, Carlsbad, Calif, USA). These cells were used for imaging by deconvolution microscopy as previously described [19, 20] for 3-dimensional projection rotational reconstruction image analysis to identify subcellular location of activated STAT5. Statistical analyses of flow cytometric data were performed using Prism 5 software (GraphPad, San Diego, Calif, USA), with pairwise comparisons being done using Student's *t-*test or Mann-Whitney U analyses and multiple group analyses done using one-way ANOVA.

### 2.4. Double chromatin immunoprecipitation (dbChIP) of chromatin-bound phospho-STAT5 protein

Five million bone marrow cells were cultured with or without 100 *μ*M Na vanadate (in DMSO, Sigma-Aldrich) for 30 minutes at 378°C/5%CO_2_. Half of the cultures +/− vanadate were then supplemented with 1000 U/mL GM-CSF, and all were incubated for an additional 90 minutes at 378°C/5%CO_2_. The cells were then fixed in situ, with 1% formaldehyde in 1xPBS (methanol-free, Sigma-Aldrich), for 10 minutes at 37°C prior to lysis with high SDS lysis buffer (Millipore-Upstate). The fixed lysates were sonicated to disrupt membranes and shear chromatin to approximately 1000 bp fragments then frozen at −80°C until analyzed. The sample was split into five 1 × 10^6^ cell aliquots for use in anti-STAT5Ptyr/anti-Histone H3 mediated double ChIP (dbChIP) protein isolations for Western blot analysis of STAT5 associated with histone/chromatin complexes. Aliquots used for dbChIP were precleared with salmon sperm DNA Protein A or G agarose beads (Millipore-Upstate), then incubated overnight at 4°C with antityrosine phosphorylated STAT5 (STAT5Ptyr) antibodies (Millipore-Upstate). After incubation, the antibody-bound chromatin complexes were precipitated using salmon sperm DNA Protein A agarose beads, and washed extensively with a series of increasing stringency buffers (low salt, high salt, LiCl, TE; ChIP reagent kit, Millipore-Upstate). A nonspecific antibody control ChIP (mouse IgG, Millipore-Upstate) and a sham IP containing no extract were used as negative controls.

ChIP extract aliquots dissociated from the beads in 1%SDS, 0.1 M bicarbonate buffer (Fisher Scientific). To both unprecipitated (total) extracts and ChIP samples, NaCl was added to a final concentration of 500 mM and the samples were incubated for 4 hours at 65°C to reverse the formaldehyde cross-links. The released anti-STAT5Ptyr-selected complexes were neutralized and reprecipitated (“double ChIP”, dbChIP) overnight at 4°C using precoupled anti-Histone H3-Protein G Salmon sperm DNA-coated beads (Millipore-Upstate). The dbChIP complexes were then washed with the same sequence of buffers as used in the first ChIP, and then boiled in 1x Leammili buffer (BioRad, Hercules, Calif, USA) for Western blot analysis. Western blots were probed with anti-STAT5 Ptyr (Millipore-Upstate) antibodies and cross-linked and uncoupled proteins were visualized using Amersham ECL plus chemilumenescence reagents (GE Healthcare/Amersham, Piscataway, NJ, USA). Densitometry analysis using BioRad Imager and Quantity One Software was used to estimate molecular weight of cross-linked dimer complexes and freed monomeric isoforms in STAT5Ptyr positive bands.

## 3. RESULTS

### 3.1. GM-CSF and M-CSF signaling interplay to regulate STAT5 activation

Mouse bone marrow cells can differentiate to mature different macrophage phenotypes within 4 days culture in M-CSF or 4–7 days in GM-CSF [3, 21, 22]. Our experiments attempted to examine myeloid differentiation at stages prior to complete maturation, at 2 to 48 hours in culture. For our *in vitro* myeloid cell differentiation experiments, NOD and C57BL/6 mouse bone marrow cells were stimulated with one cytokine (1000 U/mL GM-CSF or 500 U/mL M-CSF) in the presence of blocking antibodies for the other cytokine (anti-GM-CSF or anti-M-CSF, 2 *μ*g/mL) for 48 hours at 37°C/5%CO_2_.

**Figure 1:**
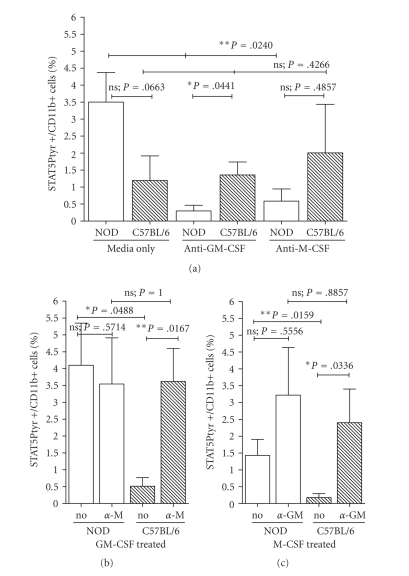
Flow cytometric analysis of STAT5 phosphorylation in *in vitro* myeloid differentiation. (a) Bone marrow cultures (1 million cells/mL) from NOD and C57BL/6 control mice were differentiated in culture with either, (b) 1000 U/mL GM-CSF plus 2*μ*g/mL anti-M-CSF blocking antibodies *α*-M), or (c) with 500 U/mL M-CSF with 2*μ*g/mL anti-GM-CSF blocking antibodies *α*-GM), for 48 hours at 3°C/5CO_2_. Cultures treated with medium only (“media only” in (a), “0” in (b) and (c)) or with medium supplemented only with antibodies to block M-CSF or GM-CSF (ANTI-M-CSF, ANTI-GM-CSF, respectively, in (a)) were run in parallel with the cytokine-stimulated cultures. In addition, separate aliquots of bone marrow cells were cultured in M-CSF or GM-CSF alone without blocking antibodies (“no” in (b) and (c)). At 48 hours, cells were stained with anti-STAT5Ptyr-FITC and anti-CD11b-PE antibodies for analysis by intracellular immune-histochemical flow cytometry. Strain and treatment regiments for each pair of cultures are listed on the *X*-axis. The percentage of STAT5Ptyr+ in CD11b+ cells is given on the *Y*-axis. Graphs are representative of 3–9 separate runs of each treatment. The *p*-values indicated were derived from Mann-Whitney U test, Student's *t*-test, or one-way ANOVA analyses, as appropriate, for the sample group comparisons indicated by brackets.

In earlier studies, we found that blocking GM-CSF signaling by anti-GM-CSF antibodies or with the Jak2/3 inhibitor AG490 in mature monocytes and macrophages did not affect the persistence of phosphorylated STAT5 [19]. In contrast, blocking endogenous GM-CSF or M-CSF stimulation by specific antibodies significantly diminished STAT5 phosphorylation in NOD bone marrow cells (*P = .0240, ANOVA, Figure 1(a)).

**Figure 2:**
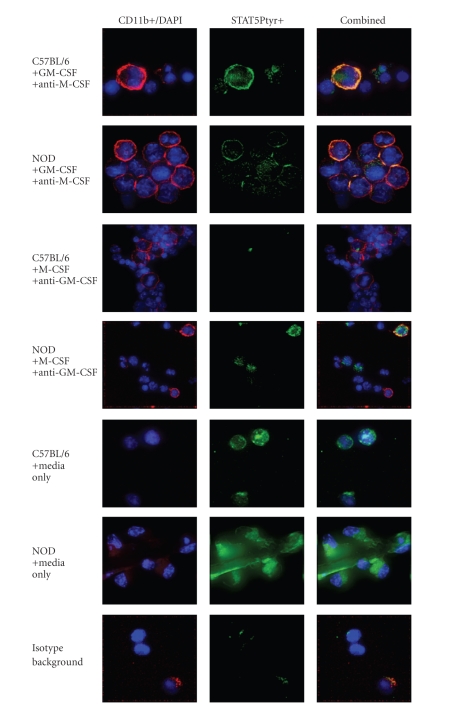
GM-CSF/M-CSF cytokine-induced myeloid differentiation *in vitro.* Bone marrow cultures from NOD and C57BL/6 control mice were differentiated in culture using regiment of cytokine and anticytokine blocking antibodies. Cells were treated with either 1000 U/mL GM-CSF plus 2*μ*g/mL anti-M-CSF blocking antibodies, with 500 U/mL M-CSF plus 2*μ*g/mL anti-GM-CSF blocking antibodies, or with media alone for 48 hours at 3°C/5CO_2_. Cells were stained with anti-STAT5Ptyr-FITC (green) and anti-CD11b-PE (red) antibodies for analysis by flow cytometry (summarized in Figure 1) and deconvolution microscopy (shown here) for the development of cells with myeloid phenotype (CD11b+) and intracellular expression of tyrosine phosphorylated STAT5. Background staining for anti-STAT5Ptyr-FITC antibody was determined by parallel sample stained with a nonspecific mouse IgG isotype control (bottom most panels). The images are composites of 3D projection of 20 optical sections (0.2 micron each) showing both immunohistochemical labels as well as DAPI (blue) poststaining of chromatin within the cells. Accompanying panels show the STAT5Ptyr-FITC staining alone (green) or CD11b-PE and DAPI staining (red/blue). Treatment regiment and the cell source strain for each pair of cultures are listed to the left of the images. Images are representative of 5 random fields observed per sample treatment and 3 separate runs of the experiment.

Deconvolution microscopic image analysis (see Figure 2) suggested that we could recreate the NOD STAT5 phosphorylation phenotype in non-autoimmune C57BL/6 cells by blocking M-CSF signaling while increasing the GM-CSF stimulation to the level seen in the NOD [19, 20]. Flow cytometric analysis also indicated that blocking endogenous M-CSF while stimulating with GM-CSF promoted STAT5 phosphorylation in C57BL/6 48-hour bone marrow cultures (*P = .0167, Mann-Whitney U test, Figure 1(b)). However, when we stimulated bone marrow cultures with M-CSF *in vitro* in the presence of blocking antibodies to GM-CSF, STAT5 phosphorylation was also stimulated in C57BL/6 cultures, and remained statistically unchanged in NOD bone marrow cells (*P = .0336 and .5556, respectively, Mann-Whitney U test, Figure 2(c)).

Taken together, these findings suggest that the interplay M-CSF and GM-CSF signaling is required to properly regulate STAT5 function, and support the role of temporally sequenced GM-CSF and M-CSF signaling on controlling STAT5 activation during myeloid cell differentiation. Furthermore, this interplay is modulated by the timing and concentration of GM-CSF stimulation early in differentiation, which appears to set the stage for STAT5 activation in later stages, regardless of further stimulation by GM-CSF or M-CSF. Because the NOD cells have higher endogenous GM-CSF production [19], this temporal signaling pattern is disrupted and leads to aberrant cell responses to GM-CSF.

### 3.2. NOD bone marrow cell DNA binding of STAT5 is increased by GM-CSF

**Figure 3:**
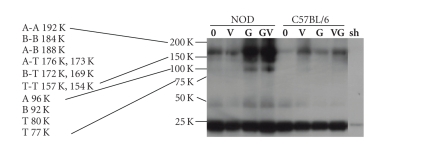
*STAT5 binding to chromatin increases after GM-CSF stimulation in NOD but not C57BL/6 bone marrow cells.* Five million bone marrow cells were cultured with or without 100*μ*M Na vanadate for 30 minutes at 37°C/5CO_2_. Half of the cultures +/− vanadate were then supplemented with 1000 U/mL GM-CSF, and all were incubated for an additional 90 minutes at 37°C/5CO_2_. The cells were then fixed *in situ*, extracted, and sonicated. The sample was split into five 1 × 10^6^ cell aliquots for use in anti-STAT5Ptyr/anti-Histone H3-mediated double ChIP protein isolations for Western blot analysis of STAT5 associated with histone/chromatin complexes. Protein isolated from the precipitated chromatin complexes and analyzed by Western blot probed with anti-STAT5Ptyr antibody. Densitometric/Rf-value anti-STAT5Ptyr Western blot-detected bands separated on 4-20% SDS-PAGE were used to give the approximate size and location of the STAT5 protein complexes and monomers (approximate molecular weights indicated on the left of the figure). Higher molecular weight bands that bound the anti-STAT5Ptyr antibody had RF values suggesting that they may represent a mix of formaldehyde-cross-linked dimer complexes containing mixed isoforms of STAT5 (STAT5A (A 96 K), STAT5B (B 92 K), and truncated (T 80 K, 77 K)), both in homodimers (A-A 192 K, B-B 184 K, T-T) and heterodimers (A-B, A-T, B-T, T-T) complexes. Only monomeric STAT5A size bands (96 K) are detected in the GM-CSF-induced NOD cell cultures at this early time point. The positions of the precipitating antibodies heavy chain (50 K) and light chain (25 K), which acts as an internal protein loading standards, were determined from the extract minus sham control (*sh*) and size standards (indicated but not shown). Treatment/lane key: 0 = untreated, V = vanadate alone, G = GM-CSF alone, VG = vanadate and GM-CSF-supplemented 2-hour cultures. Data represents 3 runs of the double ChIP analysis.

To evaluate the possibility that increased STAT5 phosphorylation could lead to increased chromatin binding in response to GM-CSF, we performed double ChIP analyses on NOD and C57BL/6 bone marrow cells that had been cultured with GM-CSF for 2 hours, and looked for *in situ* assembly of chromatin-associated protein complexes which contain both STAT5 and Histone H3 (Figure 3). We controlled for the possibility of the prolonged phosphorylation of cytoplasmic DNA-unbound STAT5 masking a more transient phosphorylation of DNA-bound STAT5 isoforms, by culturing these cells in the presence and absence of the phosphatase inhibitor, sodium vanadate. In these dbChIP analyses, we detected bands containing phosphorylated STAT5 only in GM-CSF-treated NOD cells (Figure 3). The presence or absence of the phosphatase inhibitor did not alter the banding pattern in either NOD or C57BL/6 cell extracts. Higher molecular weight STAT5-containing complexes were also detected in both NOD and C57BL/6 cell dbChIP isolates, though at markedly different levels. The level of these complexes in C57BL/6 dbChIP isolates did not significantly change with GM-CSF and/or vanadate treatment. These higher molecular weight bands may be an artifact or represent complexes containing formaldehyde cross-linked dimers of STAT5 in multiple size isoforms (i.e., STAT5A and STAT5B homo- and heterodimers, either in full-length or truncated isoforms). No bands at any of the sizes predicted for monomeric STAT5A, STAT5B, or truncated isoforms were seen associated with histone H3 in any of the treatments used in C57BL/6 cell cultures. These findings suggest that even though STAT5 can be activated in both mouse strain bone marrow cells by GM-CSF if M-CSF signaling is blocked, functional STAT5 binding on chromatin is inducible in NOD but not C57BL/6 bone marrow cells under these conditions.

## 4. DISCUSSION

Our studies indicate that overexpression of GM-CSF in both bone marrow myeloid precursor cells in NOD, but not non-autoimmune mice, can prolong the activation of STAT5 and its binding to chromatin. Furthermore, treatment of non-autoimmune mouse bone marrow cells with anti-M-CSF antibodies while stimulating them with GM-CSF at levels endogenously produced by the NOD can recreate the activation of STAT5 but not its chromatin binding. The level of STAT5 phosphorylation and function in NOD cells is independent of concurrent phosphatase or kinase [19] activity in these cells. Therefore, these findings indicate that the high level of GM-CSF produced by NOD bone marrow cells may be promoting the aberrant STAT5 activation and its potential effects on myeloid cell gene expression regulation.

Blocking GM-CSF or M-CSF signaling alone reversed the high STAT5 phosphorylation seen in the NOD, but did not alter it in non-autoimmune bone marrow cells. In addition, M-CSF treatment only significantly increased STAT5 activation in non-autoimmune cells, and only when GM-CSF signaling was concurrently blocked. These findings suggest that the regulation of STAT5 activation is affected by the interplay of GM-CSF and M-CSF signaling in differentiation, and that this interplay is disrupted in NOD myeloid cells by the persistence of GM-CSF influence on these cells.

These data leave open the question of how GM-CSF is aberrantly activated in NOD bone marrow myeloid cells in the first place. GM-CSF autocrine stimulation of myeloid cells is a key regulatory component in myeloid differentiation and activation responsiveness in mature monocytes and macrophages and is under strict temporal regulation [2, 3]. GM-CSF gene expression can be induced in myeloid cells by cytokines such as IL-6, IL-3, and IL-1*β*, which induce epigenetic control modifications on its promoter [3, 21, 23, 24]. GM-CSF downregulation prior to M-CSF activation of myeloid cells is primarily regulated through GM-CSF's own activation of IL-10 [3]. GM-CSF epigenetic regulation is accomplished through the interaction of acetylases and deacetylases at specific regions within the *Csf2* gene promoter, which STAT proteins can mediate through their function as an adaptor-DNA-binding proteins for these enzymes [ 6–25]. STAT5 can act as an adaptor for both acetylases and deacetylases [6, 7, 25]. We have found evidence that STAT5 can bind chromatin at sites previously identified as epigenetic modification sites within the *Csf2* gene promoter [26]. If such binding allows STAT5 to promote epigenetic regulation of *Csf2* gene expression, persistence of its binding at the *Csf2* promoter could promote the aberrant GM-CSF expression seen in the NOD. Furthermore, our data indicate that M-CSF signaling may be able to override NOD defects in the activation/deactivation of STAT5 related to the overproduction of GM-CSF in these cells, but its subsequent aberrant binding may be dependent on specific, unique binding site polymorphisms found within the NOD genome, especially new sites that allow STAT5 binding in the promoter of the gene for GM-CSF itself ([26] and Garrigan and Litherland, unpublished data).

The autoimmune STAT5 dysfunction seen in the NOD may be best described as a “broken switch” in epigenetic control of cytokine signaling during myeloid differentiation. In autoimmune cells, this switching mechanism is stuck in the “on” position; thereby, “shorting out” the activation of cells by the normal sequence of cytokines and blocking their proper maturation. The incompletion of myeloid cell maturation blocks the eventual functionality of this important class of APC in the initiation and maintenance of self-tolerance, which has been implicated as a major defect involved in immunopathogenesis.
